# Seed Coat Impermeability and Physical Dormancy in Amazonian *Mimosa* L. Species: Anatomical, Ecophysiological, and Germination Insights

**DOI:** 10.3390/plants15132075

**Published:** 2026-07-03

**Authors:** Maricélia Moreira dos Santos, Anderson Gustavo do Nascimento Martins, Vitor Fransuá Guedes de Sousa, José Victor Torres Alves Costa, Breno Marques da Silva e Silva

**Affiliations:** 1 Postgraduate Program in Biodiversity and Biotechnology, Rede BIONORTE, Federal University of Amapá, Macapá 68.903-419, Brazil; 2 Laboratory of Seeds and Seedlings, State University of Amapá, Macapá 68.900-070, Brazil; gustavomartir4@gmail.com (A.G.d.N.M.); v.f.guedessousa@gmail.com (V.F.G.d.S.); breno.silva@ueap.edu.br (B.M.d.S.e.S.); 3 Superintendence of Agriculture and Livestock of the State of Amapá, Macapá 68.906-380, Brazil; jose.torres@agro.gov.br

**Keywords:** Fabaceae, *Mimosa camporum*, seed anatomy, chemical scarification

## Abstract

Plants have developed several dormancy mechanisms essential for resilience in adverse environments. Understanding these mechanisms allows for the development of weed control strategies and enhances seedling production. This study aimed to investigate the anatomical and ecophysiological mechanisms associated with seed coat impermeability and physical dormancy in *Mimosa camporum* Benth. and other Amazonian *Mimosa* L. species, emphasizing their effects on water uptake, germination behavior, and ecological adaptation. For *M. camporum*, the imbibition curve, seed coat anatomy through scanning electron microscopy, and germination tests of seeds subjected to chemical scarification (H_2_SO_4_) were determined. Data from 25 Amazonian *Mimosa* species were compiled for ecological and physiological characterization, with subsequent Multiple Correspondence Analysis. Immersion in H_2_SO_4_ for 5 min is adequate to break dormancy in *Mimosa camporum* Benth. seeds. In *Mimosa camporum* Benth., sulfuric acid scarification effectively promoted water uptake and germination, demonstrating the close relationship between seed anatomy, imbibition behavior, and dormancy regulation. Physical dormancy in Amazonian *Mimosa* L. species is directly associated with seed coat impermeability, especially the presence of macrosclereids in the palisade layer. In the Amazon, the reproductive success and resilience of *Mimosa* L. species are related to the physical dormancy and desiccation tolerance of their seeds.

## 1. Introduction

Several factors are crucial for successful germination, which can be endogenous (hormonal balance, maturation, and health) and exogenous (substrate, water availability, and temperature) [1,2,3]. One of the main endogenous factors is dormancy, in which viable seeds do not germinate even under ideal conditions due to physical, morphological, physiological, or morphophysiological barriers, or a combination of these mechanisms [2,4].

Among dormancy classes, physical dormancy stands out as the second most common type, characterized mainly by seed coat impermeability caused by the palisade layer and the presence of hydrophobic substances, limiting water uptake and gas exchange [2,5,6]. This increases the species’ chances of survival, as the impermeable seed coat protects the embryo against adverse conditions (unfavorable climate, pathogen action, and others), allowing the seed to remain viable for long periods until a biotic or abiotic factor overcomes this dormancy [1,2,5,7].

Physical dormancy is notably observed in the Fabaceae family, the third largest family among angiosperms, with more than 20,000 recorded species [8,9]. One of the most notable genera of this family is *Mimosa* L. (Caesalpinioideae; Mimosoid Clade), whose more than 500 species are natively distributed mainly throughout the Americas and, to a lesser extent, in Africa and Asia [10,11,12].

*Mimosa* species are mainly used for ornamental purposes and in the timber, textile, and food industries, in addition to being widely studied for their great cosmetic and pharmacological potential [13]. Furthermore, they exhibit characteristics that make them effective for ecological restoration, such as symbiosis with nitrogen-fixing bacteria and leaf-litter production, which promote nutrient cycling [14,15]. One example is *Mimosa camporum* Benth., a pioneer herbaceous species native to the Neotropical region (especially the Amazon), very present in vegetation transition zones or areas subjected to anthropogenic intervention [16,17].

Despite their advantages, the adaptive characteristics of this genus favor its adaptation to different environments beyond its native habitat. *Mimosa diplotricha* C.Wright ex Sauvalle, *Mimosa pudica* L., and *Mimosa pigra* L. are classified as some of the worst invasive species in the world [18,19]. Their rapid spread forms a dense vegetation cover that prevents the regeneration of native plants, reducing local biodiversity [20,21,22,23]. In addition, they also cause negative impacts on agriculture, such as the blockage of irrigation systems, reduced productivity in crops such as cassava, and the poisoning/death of livestock due to nephrotoxicity [21,23,24].

Like most Fabaceae species, *M. camporum* seeds exhibit physical dormancy and require some type of scarification to germinate [17]. Chemical scarification with sulfuric acid (H_2_SO_4_) is one of the most common methods used for this genus because it promotes increased, accelerated, and/or standardized germination of impermeable seeds [6,25,26,27]. The acid acts mainly on seed coat areas that may function as water gaps, such as the lens, micropyle, hilar fissure, and/or, in many *Mimosa* species, the pleurogram [6]. Therefore, the exposure time must be determined for each species to avoid embryo damage [25].

Considering the economic and ecological importance of these species, especially in the Amazon region with the increasing extent of degraded areas, the objective of this study was to investigate the anatomical and ecophysiological mechanisms associated with seed coat impermeability and physical dormancy in *Mimosa camporum* Benth. and other Amazonian *Mimosa* L. species, emphasizing their effects on water uptake, germination behavior, and ecological adaptation.

## 2. Results

### 2.1. Dormancy in Mimosa camporum Benth. Seeds

**Figure 1:**
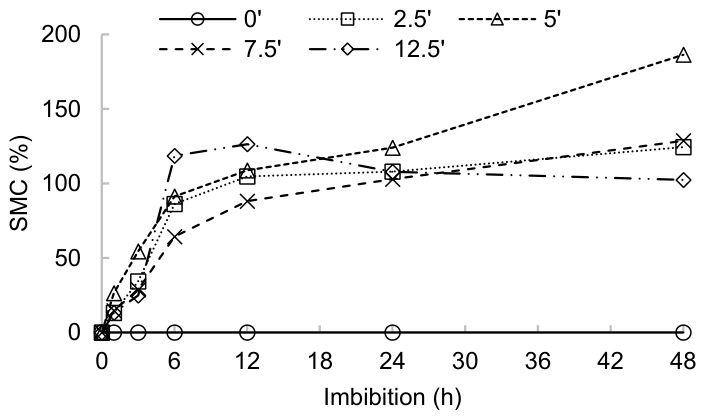
Imbibition curve of *Mimosa camporum* Benth. seeds chemically scarified with H_2_SO_4_. Legend: (′) minute, (SMC) seed moisture content, (h) hour.

Intact seeds of *M. camporum* did not absorb water after 48 h of imbibition (Figure 1). Water uptake was evaluated in seeds scarified by immersion in H_2_SO_4_ for 2.5, 5.0, 7.5, and 12.5 min, and gradually stabilized after 12 h (Figure 1). Water absorption occurred freely, exhibiting a three-phase pattern according to Bewley and Black [28], with rapid water uptake (Phase I) during the first 12 h, limited water uptake (Phase II) between 12 and 24 h, and restarted water uptake (Phase III) during the final hours (Figure 1).

**Figure 2:**
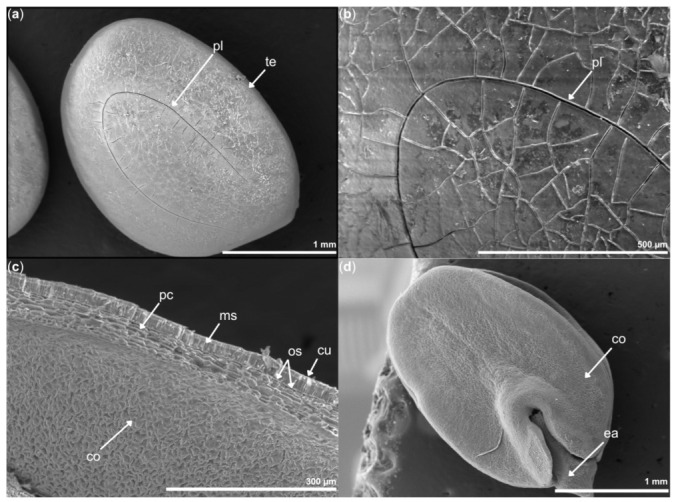
Scanning electron micrographs of *Mimosa camporum* Benth. seeds. (**a**) External view. (**b**) Details of the pleurogram. (**c**) Cross-section of the seed (seed coat and cotyledon). (**d**) Embryo. Legend: (co) cotyledon, (cu) cuticle, (ea) embryonic axis, (ms) macrosclereids, (os) osteosclereids, (pc) parenchymatic cells, (pl) pleurogram, (te) testa.

Seeds of *M. camporum* are stenospemic, bitegmic, exalbuminous and elliptical (Figure 2a), with minimum, average and maximum dimensions of 2.1, 2.6 and 3.8 mm in length, 1.5, 2.0 and 3.0 mm in width, and 0.8, 1.2 and 2.2 mm in thickness, and a dry mass of 0.003, 0.004 and 0.004 g seed^−1^.

The seed coat of *M. camporum* is glabrous, smooth, glossy, and brown, with an elliptical pleurogram; a small, homochromous hilum; a small, homochromic micropyle in relation to the seed, presenting a lateral protuberance indicating the presence of the embryonic axis (Figure 2). The seed coat consists of distinct layers: cuticle (a coating of hydrophobic substances), epidermis (with a compact palisade layer consisting of radially elongated macrosclereids with densely thickened cell walls), hypodermis (hourglass-shaped cells or osteosclereids), and parenchymatic cells (Figure 2c).

The embryo of *M. camporum* is invaginated, with free, opposite, flat, elliptical, foliaceous cotyledons with entire margins, glabrous, with smooth surfaces, and pale-yellow coloration (Figure 2d). The embryonic axis is differentiated into a short, cylindrical hypocotyl-radicle axis and a plumule with a paripinnate leaf primordium (Figure 2d).

**Figure 3:**
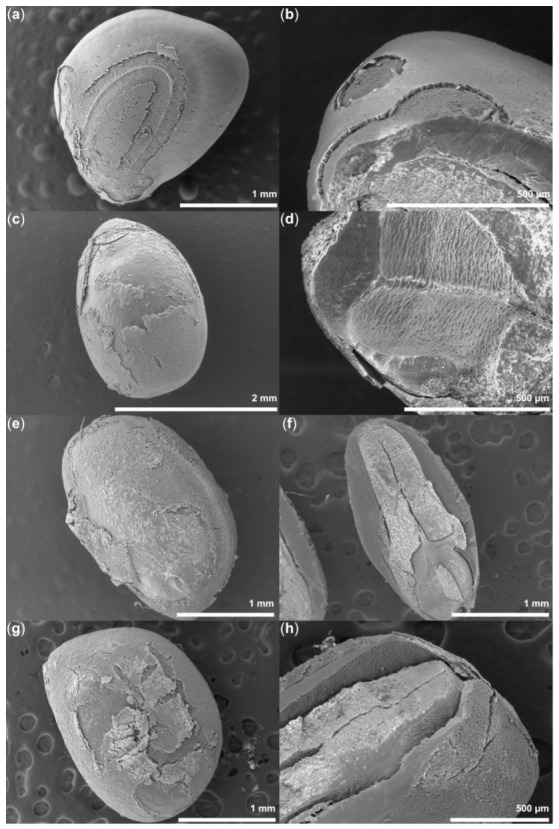
Scanning electron micrographs of *Mimosa camporum* Benth. seeds chemically scarified with H_2_SO_4_. (**a**) External view and (**b**) cross-section and after 2.5 min, showing rupture of the cuticle and epidermal corrosion; (**c**) external view and (**d**) cross-section after 5 min, showing rupture of the cuticle and epidermis; (**e**) external view and (**f**) cross-section after 7.5 min, showing rupture of the cuticle, epidermis, and hypodermis and corrosion of the parenchymatic cells of the testa; and (**g**) external view and (**h**) cross-section after 12.5 min, showing rupture of the cuticle, epidermis, and hypodermis, with subsequent rupture and corrosion of the embryo (embryonic axis and cotyledon parenchymatic cells).

In *M. camporum* seeds scarified with H_2_SO_4_ for 2.5 min, generalized cuticle corrosion (rupture) and the beginning of superficial and localized corrosion of the macrosclereids in the palisade layer of the seed coat epidermis were observed (Figure 3a,b). At 5.0 min, in addition to generalized cuticle corrosion, generalized epidermal corrosion occurred, mainly near the pleurogram, together with a slight hypodermal corrosion (Figure 3c,d). At 7.5 min, besides the rupture of the cuticle and epidermis, generalized hypodermal corrosion and corrosion of the parenchymatic cells of the seed coat and cotyledons were observed (Figure 3e,f). Finally, after 12.5 min, besides the complete degradation of the seed coat, the embryo integrity was compromised (Figure 3g,h).

**Figure 4:**
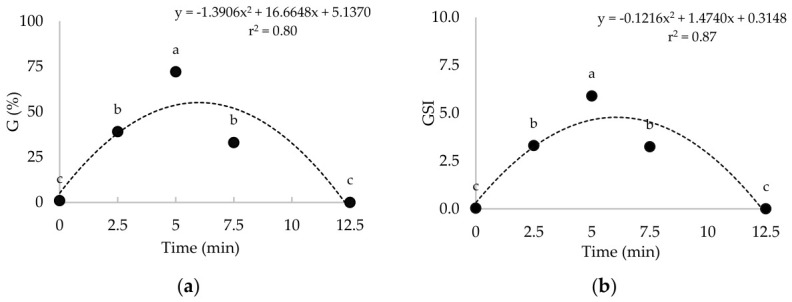
(**a**) Germination percentage (G) and (**b**) germination speed index (GSI) of *Mimosa camporum* Benth. seeds chemically scarified with H_2_SO_4_ at different exposure times (0.0, 2.5, 5.0, 7.5 and 12.5 min). Means followed by the same letter do not differ from each other by Tukey’s test (*p* < 0.05).

Seeds of *M. camporum* without scarification and those immersed in H_2_SO_4_ for 12.5 min exhibited null or nearly null germination percentage and Germination Speed Index (GSI) (Figure 4a,b). The highest germination percentage and GSI were observed at 5.0 min of immersion in H_2_SO_4_, with maximum estimated values occurring at 5.99 and 6.06 min of immersion for germination percentage and GSI, respectively (Figure 4a,b).

**Figure 5:**
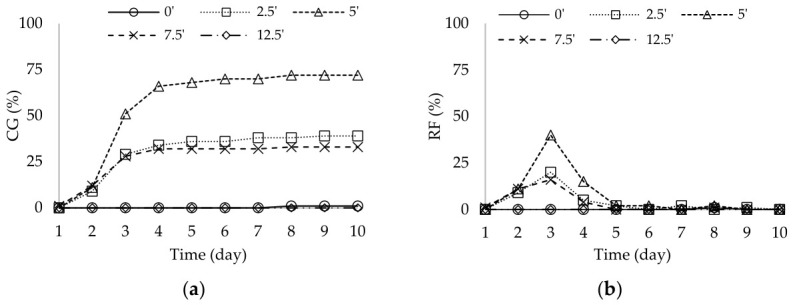
(**a**) Cumulative germination frequency (CG) and (**b**) relative germination frequency (RF) of *Mimosa camporum* Benth. seeds chemically scarified with H_2_SO_4_ at different exposure times (0.0, 2.5, 5.0, 7.5 and 12.5 min).

The greatest increase in cumulative germination percentage and the highest peak in germination frequency of *M. camporum* were observed in seeds immersed in H_2_SO_4_ for 5.0 min (Figure 5).

### 2.2. Dormancy in Amazonian Mimosa L. Seeds

In the Amazon region, most *Mimosa* species are shrubs exhibiting epigeal phanerocotylar germination with foliaceous cotyledons, craspedium-type fruits, and orthodox and impermeable seeds associated with the presence of pleurogram and/or palisade layer and/or hardness, occurring in dry environments with savanna vegetation and savanna phytophysiognomy (Table 1).

**Figure 6:**
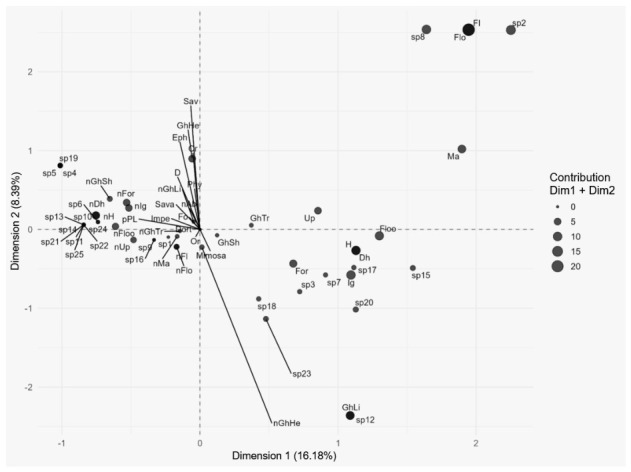
Multiple correspondence analysis between parameters and classes for Amazonian *Mimosa* L. Legend: (Dim1) Dimension 1; (Dim2) Dimension 2; *Mimosa* species: [(sp1) *M. acutistipula* var. *ferrea*); (sp2) *M. bimucronata*; (sp3) *M. caesalpiniifolia*; (sp4) *M. camporum*; (sp5) *M. carajarum*; (sp6) *M. claussenii*; (sp7) *M. daleoides*; (sp8) *M. diplotricha*; (sp9) *M. dolens*; (sp10) *M. echinocaula*; (sp11) *M. foliolosa*; (sp12) *M. invisa*; (sp13) *M. leiocephala*; (sp14) *M. orthacantha*; (sp15) *M. pigra*; (sp16) *M. pteridifolia*; (sp17) *M. pudica*; (sp18) *M. schomburgkii*; (sp19) *M. skinneri*; (sp20) *M. somnians*; (sp21) *M. somnians* var. *viscida*; (sp22) *M. spixiana*; (sp23) *M. strobiliflora*; (sp24) *M. tenuiflora*; (sp25) *M. verrucosa*]; Seed [Desiccation tolerance: (Or) Orthodox, (UnOr) Unorthodox; Dormancy: (Dort) Dormant, (nDort) Non dormant; Pleurogram/Palisade Layer: (pPL) Presence, (abPL) Absence; Seed coat: (Pe) Permeable, (Impe) Impermeable; Type of dormancy: (Phy) Physical; Type of fruit: (Cr) Craspedium; Water absorption: (Ab) Absorbs, (nAb) Does not absorb]; Seedling [Cotyledon: (Fo) Foliaceous, (nFo) Non foliaceous; Seedling type: (Eph) Epigeal phanerocotylar, (nEph) Non epigeal phanerocotylar]; Growth habit [Herbaceous: (GhHe) Yes, (nGhHe) No; Liana: (GhLi) Yes, (nGhLi) No; Shrub: (GhSh) Yes, (nGhSh) No; Tree: (GhTr) Yes, (nGhTr) No]; Environment [Dry: (D) Yes, (nD) No; Dry-humid: (Dh) Yes, (nDh) No; Humid: (H) Yes, (nH) No]; Type of vegetation [Floodplain: (Fl) Yes, (nFl) No; Forest: (For) Yes; (nFor) No; Savanna: (Sav) Yes, (nSa) No]; Phytophysiognomy [Floodplain field: (Flo) Yes, (nFlo) No; Floodplain forest: (Floo) Yes, (nFloo) No; Igapó forest: (Ig) Yes, (nIg) No; Mangrove: (Ma) Yes, (nMa) No; Savanna: (Sava) Yes, (nSava) No; Upland forest: (Up) Yes, (nUp) No].

An intersection was observed among the parameters *Mimosa* genus, dormancy presence, and physical dormancy with craspedium-type fruits, epigeal phanerocotylar germination, presence of pleurogram and/or palisade layer, impermeable seed coats, orthodox seeds, dry environment, savanna vegetation, and savanna phytophysiognomy (Figure 6).

## 3. Discussion

### 3.1. Dormancy in Mimosa camporum Benth. Seeds

Intact seeds of *M. camporum* did not absorb water freely (Figure 1). Literature data for other Amazonian species of the genus indicate that this is the standard pattern (Table 1), in which impermeability is attributed to the presence of macrosclereids in the palisade layer of the seed coat epidermis [6,24,67,68], as observed in *M. camporum* (Figure 2). Not only *Mimosa* species, but also the Fabaceae family, are characterized by physical dormancy conditioned by seed coat impermeability to water and/or gases [2,69,70].

In addition to anatomy, the chemical composition of the seed coat directly influences seed permeability. Lignin contributes to the formation of physical and chemical barriers, protecting the embryo against mechanical damage and pathogens, while also modulating permeability to water and gases [5,71,72]. Furthermore, the cell wall is composed of cellulose, hemicellulose, and hydrophobic substances such as suberin, waxes, and tannins [73,74,75]. Thus, the composition and concentration of these components modulate the physical and physiological properties of plants [76,77].

Immersion of *M. camporum* seeds in H_2_SO_4_ caused disruption of the seed coat barrier (Figure 3), enabling water uptake within the first hour (Figure 1). The effectiveness of scarification in overcoming physical dormancy has been documented for several *Mimosa* species, including *M. bimucronata* [78], *M. flocculosa* Burkart [79], *M. scabrella* Benth. [80], *M. caesalpiniifolia* [27,81,82], *M. tenuiflora* [83], *M. ophthalmocentra* Mart. ex Benth. [84], and *M. verrucosa* [66].

In *M. camporum* seeds scarified with H_2_SO_4_ for 5 min, imbibition occurred freely, following the triphasic pattern described by Bewley and Black [28]. Phase I (rapid uptake) extended through the first 12 h, followed by Phase II (limited uptake) between 12 and 24 h, while Phase III (rapid uptake again) occurred after 24 h (Figure 1). For scarified seeds of *M. ophthalmocentra*, *M. flocculosa*, *M. caesalpiniifolia*, and *M. verrucosa*, Phases I, II, and III began, respectively, at intervals of 1, 7, and 19 h; 1, 6, and 11 h; 2, 12, and 36 h; and 1, 8, and 16 h [66,79,82,84].

Morphologically, the seed coat of *M. camporum* is glabrous, smooth, glossy, and brown. The pleurogram is elliptical, with small hilum and micropyle homochromous with the seed. Anatomically, the seed coat consists of four distinct layers: cuticle (a coating of hydrophobic substances), epidermis (a compact palisade layer composed of radially elongated macrosclereids with densely thickened cell walls), hypodermis (osteosclereids), and parenchymatic cells (Figure 2c).

This anatomical structure resembles that described for other species of the genus, such as *M. flocculosa* and *M. pudica* [6,68]. The presence of a lipidic cuticle and macrosclereids with a lipidic matrix in the palisade layer has also been reported in other Fabaceae species [85,86]. The anatomy and chemistry of the seed coat, especially the cuticle and palisade layer of the seed coat epidermis, determine impermeability and regulate water uptake [1,2,85,86].

During the 48 h water uptake period, seeds subjected to scarification for 2.5, 7.5, and 12.5 min had lower water content than those treated for 5.0 min (Figure 1). In *M. camporum* seeds scarified with H_2_SO_4_ for 2.5 min, generalized cuticle rupture and the beginning of superficial erosion of macrosclereids in the palisade layer were observed (Figure 3a,b). At 5.0 min, in addition to cuticle removal, generalized epidermal rupture occurred, especially near the pleurogram, reaching the hypodermis (Figure 3c,d). At 7.5 min, degradation advanced into the hypodermis, parenchymatic cells, and cotyledonary tissues (Figure 3e,f). Finally, at 12.5 min, total seed coat degradation resulted in loss of embryo integrity (Figure 3g,h).

Sulfuric acid is widely used to overcome physical dormancy in Fabaceae seeds, and immersion time is the determining factor for the success of the technique. Insufficient exposure periods do not adequately disrupt the seed coat, whereas prolonged immersion may compromise embryo integrity, rendering the seeds nonviable. Therefore, the efficiency of the technique is directly associated with the definition of an optimal exposure time that maximizes germination while preserving embryonic tissues [2,5,87].

In this sense, the highest germination percentage and GSI of *M. camporum* seeds were observed after 5.0 min of immersion in H_2_SO_4_. Maximum values for germination percentage and GSI were estimated at 5.99 and 6.06 min, respectively (Figure 4). Beyond these points, both variables decreased quadratically until reaching a minimum at 12.28 min, when embryo integrity was compromised (Figure 4). Several studies have demonstrated that the optimal immersion time for breaking dormancy in *Mimosa* seeds ranges from 1 to 60 min [35,36,66,68,79,88,89,90,91,92,93,94,95,96,97], reflecting differences in seed coat thickness, cell types, and lipidic matrices among Fabaceae species [5,6,68,86].

Immersion of *M. camporum* seeds in H_2_SO_4_ for 5.0 min promoted the highest germination percentage (72%) (Figure 3a). Similar results were reported for *M. bimucronata*, in which immersion in H_2_SO_4_ for 5 and 10 min produced the most satisfactory results among several scarification methods tested [35]. Similarly, *M. verrucosa* showed 81% germination after 10 min of acid exposure [66]. In contrast, for *M. pudica*, the treatment efficiency extended over longer intervals, where times of 1, 5, 15, and 20 min were statistically similar, exceeding 80% germination [97].

Montaño-Arias et al. [98] recommended mechanical scarification for *Mimosa* species to achieve high germination rates. However, for *M. camporum*, the present study demonstrated statistically superior performance of chemical scarification (72%) compared with the mechanical abrasion reported by Maciel et al. [17] (62 ± 7.48%). In addition, manual handling of the seeds is difficult and uniform mechanical abrasion is limited due to their small size, making sulfuric acid treatment a more efficient and consistent alternative. Regardless of the method, both processes promote seed coat rupture, favoring imbibition and gas exchange essential for germination [99].

The distribution of relative germination frequency showed a unimodal configuration (Figure 5b), consistent with patterns observed in *M. caesalpiniifolia* germinated on paper regardless of temperature [100]. Germination intensified on the third day after experiment establishment, reaching stability from the fifth day onward (Figure 5a), especially in the treatment involving immersion in H_2_SO_4_ for 5 min.

These results reflect the increase in GSI following immersion in H_2_SO_4_. GSI is one of the most important concepts related to seed vigor, as seeds with identical final germination percentages may differ significantly in their rate of establishment [101]. In the context of chemical scarification, GSI may also reflect the extent of damage caused by the chemical agent to the embryo and its nutrient reserves [5].

This phenomenon became evident after 7.5 min of exposure (3.24 ± 0.78), when a marked reduction in GSI was observed compared with the 5 min treatment (5.89 ± 0.62), the most effective. After 12.5 min of exposure, no germination occurred, indicating toxicity (Figure 3g,h). In contrast, Maciel et al. [17] reported a GSI of 14.6 ± 2.98 for the same species using mechanical abrasion, a higher value than that obtained in this study with chemical scarification.

Seed coat deterioration by scarification increases permeability, allowing water diffusion through parenchymatic cells toward the cotyledon periphery and embryo hydration [17,102]. However, rapid water uptake does not always imply a positive response, since it may cause cellular damage, electrolyte leakage, and other conditions that negatively affect germination [102].

This latter behavior was observed in the 12.5 min treatment, in which greater seed coat degradation resulted in higher water content than in the other treatments between 6 and 12 h of imbibition (Figure 1). After this period, this was the only group showing a decline in water content. This decrease is related to embryo damage caused by scarification. Furthermore, this stress started enzymatic degradation, altering the consistency of the embryonic structure from firm to gelatinous, with subsequent fungal and bacterial proliferation. This explains the apparent reduction in water content due to the loss of embryo content and/or parts, rather than an actual loss of water.

Unlike *M. luisana*, in which intact and scarified seeds absorbed 80% of their total water uptake capacity within 3 h [103], the optimized treatment for *M. camporum* required approximately 6 h to reach the same hydration level (Figure 1). No water uptake was observed in intact seeds of *M. camporum*, confirming the effectiveness of chemical scarification in overcoming seed coat impermeability (Figure 1).

### 3.2. Dormancy in Amazonian Mimosa L. Seeds

In the Amazon region, *Mimosa* species are predominantly shrubs occurring in dry environments, savanna vegetation, and savanna phytophysiognomy (Table 1). Germination is typically epigeal phanerocotylar with foliaceous cotyledons; fruits are predominantly craspedium-type, and seeds are orthodox and impermeable, associated with the presence of a pleurogram and/or palisade layer (Table 1). According to Flora e Funga do Brasil [29] and speciesLink [49], most Amazonian *Mimosa* species are savanna shrubs with seeds tolerant to desiccation and physical dormancy associated with seed coat impermeability [6,104,105].

In the Multiple Correspondence Analysis (MCA), the spatial distribution, particularly in the central intersection, associated the genus *Mimosa* with physical dormancy (seed coat permeability and anatomy) and savanna phytophysiognomy (dry and savanna environments) (Figure 6). This reflects a set of functionally integrated traits favoring persistence, colonization, and responses to recurrent environmental disturbances in savannas, such as fire, smoke, and drought [44,51,106,107,108,109].

Leguminous species characteristic of seasonally dry environments frequently exhibit physical dormancy resulting from the layer of lignified palisade cells and deposition of hydrophobic substances [2,85,110]. The high incidence of this characteristic represents an evolutionary response to the climatic unpredictability of these environments, in which persistence depends on soil seed banks and germination conditioned by environmental triggers such as temperature fluctuations, water availability, and fire, among others [2,106,107,109].

For *Mimosa*, physical dormancy is an essential adaptive mechanism for survival in seasonal environments because it regulates germination and prevents seedling emergence under unfavorable conditions, such as water deficit [6,44,111,112]. Furthermore, the relationship between savanna phytophysiognomies and physical dormancy in Fabaceae species is common, reinforcing the pattern observed in the MCA (Figure 6) as a reflection of evolutionarily conserved ecological strategies [2,44].

In savannas, the integration among physical dormancy, fruit morphology, and seedling characteristics constitutes a functional axis in plant community structuring, influencing regeneration, establishment, and persistence strategies under seasonal water limitation [108,113,114]. In this context, the craspedium-type fruit, characteristic of several *Mimosa* species, favors protection and gradual release of seeds and, when associated with physical dormancy, optimizes establishment when environmental conditions are favorable [6,44,115].

Simultaneously, the orthodox nature of *Mimosa* seeds confers high desiccation tolerance, allowing seeds to remain viable even under low water content and high temperatures, thus ensuring the persistence of the soil seed bank [108]. Within the genus *Mimosa*, such characteristics are frequent because many species occupy seasonally dry environments and possess hard, impermeable seed coats associated with physical dormancy and high desiccation tolerance. This set of attributes regulates germination in response to moisture pulses, such as the onset of rainfall, ensuring persistence during adverse periods [116].

## 4. Materials and Methods

### 4.1. Collection of Plant Material

Fruits and seeds of *M. camporum* were collected in a savanna area in Macapá, Amapá, Amazon, Brazil (0°03′33.5″ N 51°05′15.5″ W). The species survey, literature review, and both descriptive and statistical seed analyses were conducted at the Laboratory of Seeds and Seedlings, State University of Amapá (UEAP), Macapá, Brazil, and the Laboratory of Drug Research, Federal University of Amapá (UNIFAP), Macapá, Brazil. For the biometric characterization, four replicates of 25 seeds were measured using calipers (0.05 mm precision). Additionally, to determine seed weight, 100 seeds were weighed individually using an analytical balance (0.0001 g precision).

According to the Köppen–Geiger climate classification, the climate in Macapá is classified as Aw, corresponding to a tropical climate characterized by two distinct seasons: winter (“Inverno Amazônico”) (December to August), with peak rainfall occurring in March, and summer (“Verão Amazônico”) (September to November), characterized by high temperatures and low precipitation [117,118]. The phytophysiognomy of the urban area of Macapá consists predominantly of *cerrado* vegetation (savanna), with dispersed woody flora adapted to adverse environmental conditions and resistant to seasonal fires [119]. The soils in the region are predominantly classified as Yellow Latosol (Oxisol), characterized by low natural fertility [119].

### 4.2. Dormancy in Mimosa camporum Benth. Seeds

The fruits were dried in the shade for 24 h and manually processed by twisting the legumes. Only intact seeds were selected, excluding those visibly predated. To overcome physical dormancy, groups of 100 seeds per treatment were immersed in sulfuric acid (H_2_SO_4_, ACS grade, 98% purity, Impex^®^ Analytical Reagents, São Paulo, Brazil) at a ratio of 10 mL of acid per group to ensure full submersion. The exposure periods evaluated were 0, 2.5, 5, 7.5, and 12.5 min. Immediately after each period, the acid was drained, and the seeds were rinsed three times in 50 mL of distilled and deionized water (5 min per rinse) under constant agitation to ensure the complete removal of chemical residues.

The imbibition curve was determined using three replicates of 10 seeds per treatment. Seeds were placed in Petri dishes (10 cm) on two sheets of germination paper moistened with distilled and deionized water (2.5 times the dry weight of the paper) and maintained at 30 °C [120,121]. Seed mass measurements were obtained using an analytical balance (0.0001 g precision) at intervals of 0, 1, 3, 6, 12, 24, and 48 h. The SMC was calculated according to the following equation [122]:

SMC = [(Fm − Pi)/Pi] × 100(1)

where SMC is the seed moisture content (water absorption percentage), Fm is the fresh mass of the seeds after immersion and Pi is the initial fresh mass of the seeds before immersion.

For the description of seed morphology and seed coat anatomy before and after chemical scarification, whole seeds and seeds transversely sectioned using a steel blade were utilized [123]. The samples were subsequently analyzed using a tabletop scanning electron microscope (HITACHI^®^ TM3030Plus, São Paulo, Brazil) [123].

The germination test was conducted using four replicates of 25 seeds placed in Petri dishes (10 cm) on two sheets of germination paper moistened with distilled and deionized water (2.5 times the dry weight of the paper) and maintained at 30 °C under a 12 h photoperiod [121].

Daily counts were performed at the same hour for 10 days. Germination percentage was calculated according to the following equation [124]:

G = (Σn_i_/N) × 100 (2)

where G is the germination percentage, n_i_ is the total number of germinated seeds on day i, and N is the total number of seeds placed to germinate. GSI was calculated according to the following equation [125]:

GSI = Σ(n_i_/t_i_) (3)

where GSI is germination speed index, n_i_ is the number of seeds germinated at time i, and ti is the time passed from the beginning of the test. The relative germination frequency was calculated according to the following equation [126]:

RF = n_i_/Σn_i_ (4)

where RF is the relative germination frequency, n_i_ is the total number of germinated seeds on day i, and Σn_i_ is the total number of germinated seeds. The cumulative germination frequency is the sum of the relative frequencies over the period of the experiment.

The experimental design was completely randomized. Data were subjected to analysis of variance (ANOVA), and when significant according to the F test, means were compared using Tukey’s test (*p* < 0.05). Regression equations were fitted for the seed immersion times in H_2_SO_4_. Statistical analyses were performed using the vegan package in R software (version 4.5.3) [127,128].

### 4.3. Dormancy in Amazonian Mimosa L. Seeds

According to Checklist of the Plants of the Guianas [39,40], Plants of the World Online [33], Flora e Funga do Brazil [29], speciesLink [49], Atlas of Living Australia [32], and C. V. Starr Virtual Herbarium [31], 118 species of *Mimosa* have been recorded in the Amazon region. Following a review of online herbarium databases, including speciesLink [49] and C. V. Starr Virtual Herbarium [31], as well as exsiccates available from HAMAB, an herbarium belonging to the Institute of Scientific and Technological Research of the State of Amapá (IEPA), 25 species presenting 100% of the required information were selected.

The 13 qualitative characters were defined by the authors and comprised environmental, phytophysiognomic, ecological, morphological, and physiological information related to the seeds/seedlings. The following parameters and classifications were evaluated: 1. Fruit type (Fabaceae): legume, moniliform, follicle, nucoid, bacaceous, craspedium, loment, samara, samaroid, drupaceous, cryptoloment, and cryptosamara [129]; 2. Pleurogram: presence or absence [130]; 3. Water uptake: yes or no [1]; 4. Seed coat: permeable or impermeable [1]; 5. Desiccation tolerance: orthodox and unorthodox (intermediate or recalcitrant) [1]; 6. Dormancy: presence or absence [2]; 7. Dormancy type: non-dormant, physiological dormancy, physical dormancy, morphological dormancy, morphophysiological dormancy, and combined dormancy [2]; 8. Seedling type: epigeal phanerocotylar or non-epigeal phanerocotylar (epigeal cryptocotylar, hypogeal cryptocotylar, or hypogeal phanerocotylar) [131]; 9. Cotyledon type: foliaceous or storage cotyledons [132]; 10. Growth habit: tree, shrub, herbaceous and liana [131]; 11. Environment: dry, humid or humid-dry [133]; 12. Vegetation type (homogeneity): floodplain, forest or savanna [133]; and 13. Phytophysiognomy: savanna, floodplain field, upland forest, transition forest, floodplain forest, igapó forest and mangrove [133,134].

Initially, percentages were calculated for the parameters described above. Subsequently, the joint relationship between dormancy occurrence and the qualitative characters was explored through MCA, aiming to understand the relationships among qualitative variables based on spatial proximity [135,136,137]. In MCA, analyses are performed using multidimensional tables in which rows represent observations (species) and columns represent the different categories of distinct variables [138,139]. This method was selected due to the nature of the data (categorical) and the large number of variables (qualitative characters), and analyses were performed in R software using the FactoMineR and factoextra 2.0.0 packages [128,140,141].

## 5. Conclusions

Immersion in H_2_SO_4_ for 5 min is adequate to break dormancy in *Mimosa camporum* Benth. seeds.

In *Mimosa camporum* Benth., sulfuric acid scarification effectively promoted water uptake and germination, demonstrating the close relationship between seed anatomy, imbibition behavior, and dormancy regulation.

Physical dormancy in Amazonian *Mimosa* L. species is directly associated with seed coat impermeability, especially the presence of macrosclereids in the palisade layer.

In the Amazon, the reproductive success and resilience of *Mimosa* L. species are related to the physical dormancy and desiccation tolerance of their seeds.

## Figures and Tables

**Table 1:** Morphology, anatomy, water uptake, dormancy types, and ecology of Amazonian *Mimosa* L. seeds.

***Mimosa*** **Species**					**Seed**																		**Seedling**		
					**Tf**			**PL**			**Wa**	**Coat**			**Dt**			**Dor**		**TDor**			**St**		**Co**
*M. acutistipula var. ferrea* Barneby					Cr			pPL			nAb	Impe			Or			Dort		Phy			Eph		Fo
*M. bimucronata* (DC.) Kuntze					Cr			pPL			nAb	Impe			Or			Dort		Phy			Eph		Fo
*M. caesalpiniifolia* Benth.					Cr			pPL			nAb	Impe			Or			Dort		Phy			Eph		Fo
*M. camporum* Benth.					Cr			pPL			nAb	Impe			Or			Dort		Phy			Eph		Fo
*M. carajarum* (Barneby) T.P.Mendes & M.J.Silva					Cr			pPL			nAb	Impe			Or			Dort		Phy			Eph		Fo
*M. claussenii* Benth.					Cr			pPL			nAb	Impe			Or			Dort		Phy			Eph		Fo
*M. daleoides* Benth.					Cr			pPL			nAb	Impe			Or			Dort		Phy			Eph		Fo
*M. diplotricha* C.Wright ex Sauvalle					Cr			pPL			nAb	Impe			Or			Dort		Phy			Eph		Fo
*M. dolens* Vell.					Cr			pPL			nAb	Impe			Or			Dort		Phy			Eph		Fo
*M. echinocaula* Benth.					Cr			pPL			nAb	Impe			Or			Dort		Phy			Eph		Fo
*M. foliolosa* Benth.					Cr			pPL			nAb	Impe			Or			Dort		Phy			Eph		Fo
*M. invisa* Mart. ex Colla					Cr			pPL			nAb	Impe			Or			Dort		Phy			Eph		Fo
*M. leiocephala* Benth.					Cr			pPL			nAb	Impe			Or			Dort		Phy			Eph		Fo
*M. orthacantha* Benth.					Cr			pPL			nAb	Impe			Or			Dort		Phy			Eph		Fo
*M. pigra* L.					Cr			pPL			nAb	Impe			Or			Dort		Phy			Eph		Fo
*M. pteridifolia* Benth.					Cr			pPL			nAb	Impe			Or			Dort		Phy			Eph		Fo
*M. pudica* L.					Cr			pPL			nAb	Impe			Or			Dort		Phy			Eph		Fo
*M. schomburgkii* Benth.					Cr			pPL			nAb	Impe			Or			Dort		Phy			Eph		Fo
*M. skinneri* Benth.					Cr			pPL			nAb	Impe			Or			Dort		Phy			Eph		Fo
*M. somnians* Humb. & Bonpl. ex Willd.					Cr			pPL			nAb	Impe			Or			Dort		Phy			Eph		Fo
*M. somnians var. víscida* (Willd.) Barneby					Cr			pPL			nAb	Impe			Or			Dort		Phy			Eph		Fo
*M. spixiana* Barneby					Cr			pPL			nAb	Impe			Or			Dort		Phy			Eph		Fo
*M. strobiliflora* Burkart					Cr			pPL			nAb	Impe			Or			Dort		Phy			Eph		Fo
*M. tenuiflora* (Willd.) Poir.					Cr			pPL			nAb	Impe			Or			Dort		Phy			Eph		Fo
*M. verrucosa* Benth.					Cr			pPL			nAb	Impe			Or			Dort		Phy			Eph		Fo
***Mimosa* Species**	**Growth Habit**									**Environment**									**Type of Vegetation**						
	**TGhTr**	**TGhSh**		**TGhHe**			**TGhLi**			**AmD**			**AmH**			**AmDh**			**TvSa**			**TvFo**		**TvFl**	
*M. acutistipula var. ferrea*	GhTr	GhSh		nGhHe			nGhLi			D									Sav			For			
*M. bimucronata*	GhTr	GhSh		nGhHe			nGhLi			D			H			Dh			Sav					Fl	
*M. caesalpiniifolia*	GhTr	GhSh		nGhHe			nGhLi			D			H			Dh			Sav						
*M. camporum*	nGhTr	nGhSh		GhHe			nGhLi			D									Sav						
*M. carajarum*	nGhTr	nGhSh		GhHe			nGhLi			D									Sav						
*M. claussenii*	GhTr	GhSh		nGhHe			nGhLi			D									Sav						
*M. daleoides*	GhTr	GhSh		nGhHe			nGhLi			D			H			Dh			Sav			For			
*M. diplotricha*	nGhTr	GhSh		GhHe			nGhLi			D			H			Dh			Sav			For		Fl	
*M. dolens*	nGhTr	GhSh		nGhHe			nGhLi			D									Sav			For			
*M. echinocaula*	nGhTr	GhSh		nGhHe			nGhLi			D									Sav						
*M. foliolosa*	nGhTr	GhSh		nGhHe			nGhLi			D									Sav						
*M. invisa*	nGhTr	GhSh		nGhHe			GhLi			D			H			Dh			Sav			For			
*M. leiocephala*	nGhTr	GhSh		nGhHe			nGhLi			D									Sav						
*M. orthacantha*	nGhTr	GhSh		nGhHe			nGhLi			D									Sav						
*M. pigra*	nGhTr	GhSh		nGhHe			nGhLi			D			H			Dh			Sav			For			
*M. pteridifolia*	nGhTr	GhSh		nGhHe			nGhLi			D									Sav			For			
*M. pudica*	nGhTr	GhSh		GhHe			nGhLi			D			H			Dh			Sav			For			
*M. schomburgkii*	GhTr	nGhSh		nGhHe			nGhLi			D			H			Dh			Sav			For			
*M. skinneri*	nGhTr	nGhSh		GhHe			nGhLi			D									Sav						
*M. somnians*	nGhTr	GhSh		nGhHe			nGhLi			D			H			Dh			Sav			For			
*M. somnians var. viscida*	nGhTr	GhSh		nGhHe			nGhLi			D									Sav						
*M. spixiana*	nGhTr	GhSh		nGhHe			nGhLi			D									Sav						
*M. strobiliflora*	nGhTr	GhSh		nGhHe			nGhLi			D			H			Dh			Sav			For			
*M. tenuiflora*	GhTr	GhSh		nGhHe			nGhLi			D									Sav						
*M. verrucosa*	nGhTr	GhSh		nGhHe			nGhLi			D									Sav						
***Mimosa* Species**	**Phytophysiognomy**																				**All** **References**				
	**PhySav**		**PhyFlo**			**PhyUp**			**PhyFloo**					**PhyIg**			**PhMa**								
*M. acutistipula var. ferrea*	Sava					Up															[16,29,30]				
*M. bimucronata*	Sava		Flo			Up			Floo					Ig			Ma				[26,29,31,32,33,34,35]				
*M. caesalpiniifolia*	Sava								Floo					Ig							[29,31,36,37,38]				
*M. camporum*	Sava																				[16,29,30,31,33,39,40,41]				
*M. carajarum*	Sava																				[16,29,42]				
*M. claussenii*	Sava																				[29,43,44]				
*M. daleoides*	Sava					Up			Floo												[29,31,32,33,45,46]				
*M. diplotricha*	Sava		Flo			Up			Floo												[6,29,31,33,39,40,47,48]				
*M. dolens*	Sava					Up															[29,33,49,50]				
*M. echinocaula*	Sava																				[29,49,51]				
*M. foliolosa*	Sava																				[29,49,51,52]				
*M. invisa*	Sava								Floo					Ig							[29,33,39,47,53,54,55]				
*M. leiocephala*	Sava																				[29,49,51]				
*M. orthacantha*	Sava																				[29,31,32,33,45]				
*M. pigra*	Sava					Up			Floo					Ig			Ma				[29,31,32,33,40,43,55,56]				
*M. pteridifolia*	Sava					Up															[29,43,51]				
*M. pudica*	Sava					Up			Floo					Ig							[6,29,30,31,32,33,39,40,55,57]				
*M. schomburgkii*	Sava													Ig							[29,31,33,39,40,49,58]				
*M. skinneri*	Sava																				[17,29,32,33,43]				
*M. somnians*	Sava					Up			Floo					Ig							[31,32,33,39,43,51]				
*M. somnians var. viscida*	Sava																				[16,29,31,40,59]				
*M. spixiana*	Sava																				[29,44,49]				
*M. strobiliflora*	Sava													Ig							[29,60,61]				
*M. tenuiflora*	Sava																				[29,31,32,33,62,63,64,65]				
*M. verrucosa*	Sava																				[29,49,66]				

Blank spaces: absence of the species in the environment, type of vegetation, and/or phytophysiognomy. Legend: Seed [(Tf) Type of fruit: (Cr) Craspedium; (PL) Pleurogram/Palisade Layer: (pPL) Presence; (Wa) Water absorption: (Ab) Absorbs, (nAb) Does not absorb; (Coat) Seed coat: (Pe) Permeable, (Impe) Impermeable; (Dt) Desiccation tolerance: (Or) Orthodox, (UnOr) Unorthodox; (Dor) Dormancy: (Dort) Dormant; (TDor) Type of dormancy: (Phy) Physical]; Seedling [(St) Seedling type: (Eph) Epigeal phanerocotylar; (Co) Cotyledon: (Fo) Foliaceous]; Growth habit [(TGhTr) Tree: (GhTr) Presence, (nGhTr) Absence; (TGhSh) Shrub: (GhSh) Presence, (nGhSh) Absence; (TGhHe) Herbaceous: (GhHe) Presence, (nGhHe) Absence; (TGhLi) Liana: (GhLi) Presence, (nGhLi) Absence]; Environment [(AmD) Dry: (D) Yes; (AmH) Humid: (H) Yes; (AmDh) Dry-humid: (Dh) Yes]; Type of vegetation [(TvSa) Savanna: (Sav) Yes; (TvFo) Forest: (For) Yes; (TvFl) Floodplain: (Fl) Yes]; Phytophysiognomy [(PhySav) Savanna: (Sava) Yes; (PhyFlo) Floodplain field: (Flo) Yes; (PhyUp) Upland forest: (Up) Yes; (PhyFloo) Floodplain forest: (Floo) Yes; (PhyIg) Igapó forest: (Ig) Yes; (PhMa) Mangrove: (Ma) Yes].

## Data Availability

The original contributions presented in this study are included in the article. Further inquiries can be directed to the corresponding author.
